# Does adaptation to vertebrate codon usage relate to flavivirus emergence potential?

**DOI:** 10.1371/journal.pone.0191652

**Published:** 2018-01-31

**Authors:** Nicholas Di Paola, Caio César de Melo Freire, Paolo Marinho de Andrade Zanotto

**Affiliations:** 1 Laboratory of Molecular Evolution and Bioinformatics, Department of Microbiology, Biomedical Sciences Institute, University of Sao Paulo, Sao Paulo, Brazil; 2 Department of Genetics and Evolution, UFSCar—Federal University of Sao Carlos, Sao Carlos, Brazil; Washington University, UNITED STATES

## Abstract

Codon adaptation index (CAI) is a measure of synonymous codon usage biases given a usage reference. Through mutation, selection, and drift, viruses can optimize their replication efficiency and produce more offspring, which could increase the chance of secondary transmission. To evaluate how higher CAI towards the host has been associated with higher viral titers, we explored temporal trends of several historic and extensively sequenced zoonotic flaviviruses and relationships within the genus itself. To showcase evolutionary and epidemiological relationships associated with silent, adaptive synonymous changes of viruses, we used codon usage tables from human housekeeping and antiviral immune genes, as well as tables from arthropod vectors and vertebrate species involved in the flavivirus maintenance cycle. We argue that temporal trends of CAI changes could lead to a better understanding of zoonotic emergences, evolutionary dynamics, and host adaptation. CAI appears to help illustrate historically relevant trends of well-characterized viruses, in different viral species and genetic diversity within a single species. CAI can be a useful tool together with *in vivo* and *in vitro* kinetics, phylodynamics, and additional functional genomics studies to better understand species trafficking and viral emergence in a new host.

## Introduction

When a virus first jumps into a human host from a non-human vector or reservoir host, conserved changes in viral population sequences can occur that could allow the virus to adapt to a new host [1,2]. Part of host adaptation relies on coping with a host’s cellular environment, in particular, the translational machinery that will allow a virus to complete its replication cycle. Codon-bias analyses have proven to be effective in extrapolating information from a zoonotic virus infection in a new, vector or reservoir host [3–8]. During viral replication, the coding regions of viral genomes undergo both mutational and selective pressures that result in non-synonymous and synonymous changes that may or may not result in adaptive changes. Codon adaptation index (CAI) quantifies how well the codon preferences of the virus match that of the host [4,9]. For example, a host cell that uses more frequently AGA to code for Arginine will generally have a greater pool of tRNAs for AGA in its cellular environment, as opposed to a rarer, less used codon for Arginine such as CGU. Although cellular conditions are “tailored” for the host’s translational needs, a virus could optimize its replicative and translational kinetics by selecting for codons more abundant in the host’s cellular environment. Although it has been shown that the translational efficiency of some persistently infecting viruses is reduced to diminish protein expression to avoid immune surveillance [7], the opposite has been observed with acutely infecting viruses.

The availability of codon usage tables for a host’s constitutively expressed or highly expressed genes could allow analysis of adaptive changes of acutely infecting zoonotic viruses [2], as they interact with new hosts. Convergence or divergence of viral codon adaptation towards or away from that of host can be observed over relatively short periods of time (*i*.*e*., years), allowing side by side comparisons with their epidemic history. There is plausibility for increased CAI and the occurrence of human disease from acutely infecting zoonotic viruses. It can be argued that a virus, whose codon usage is calibrated to the host’s usage, may have the capability to translate and replicate faster than a virus that has a lower codon adaptation to a specific host [10,11]. Because higher replication tends to increase viral titers, higher host viral load could allow for an increased chance of secondary transmission, virulence and higher chance of mortality from the infection [12].

A requirement for flaviviruses, and arborviruses in general, is the initial need to adapt to the codon choices of a vector species before the virus adapts to the codon usage choices of humans [13]. The alternation between mammals and arthropods impose constraints on arbovirus evolution. Specifically in the vector, albeit strong purifying selection, we can observe silent selection, that is changes in 4-fold degenerate codon sites, promoting differential codon usages in viral genomes [14–16].

Yellow Fever virus (YFV), Dengue virus (DENV) and West Nile virus (WNV) are globally prevalent flaviviruses that cause morbidity and mortality in humans. These three flaviviruses have extensive genetic and biological diversity within themselves, *i*.*e*. sylvatic and endemic strains of DENV and YFV, or the distinct genetic lineages of WNV [17,18], which vary in reservoir hosts and ability to infect humans in distinct biological behaviors [19–29]. To provide insights into the usage of temporally scaled CAI of YFV, DENV, and YFV in zoonotic emergences, we have performed a large analysis investigating different flavivirus vector-host relationships. We first created two codon usage tables for specific human genes that could play a role in viral codon adaptation. Next, we looked for broad codon adaptation patterns within the flavivirus genus subgroups. We used an in-depth approach to investigate the codon adaption of YFV, Dengue 2 virus (DENV-2), and WNV to known hosts by using a large set of serially stamped sequences from widespread geographical origins. Our understanding of how viral species modulate translation and adapt to host cellular environments over time is limited. From these data, we show that flaviviruses have distinct patterns of codon adaption linked vector-host subgroups and biology, and we provide evidence that species-specific codon adaptation is observable and possibly linked to the vector-host evolution across time in certain flaviviruses.

## Materials and methods

### Sequence databases

For each virus (Yellow Fever virus, Dengue 2 virus, Tobacco Mosaic virus, West Nile virus), all available complete genome sequences with collection date and country of isolation information were downloaded from Genbank. Open reading frames of sequences were exported aligned using MAFFT 7.222 [30] and then manually curated using the Geneious 9.0.5 software (www.geneious.com). See S1 Dataset for complete sequence information (Accession number, virus, collection date, country and lineage).

### Codon adaptation indices of viral coding genes

To investigate if our selected viruses have evidence for codon adaptation in humans, we calculated the codon adaptation indices (CAI) for each coding region of each respective virus. For Yellow Fever virus, Dengue 2 virus, and West Nile virus, the complete polyprotein was used to calculate CAI. For Tobacco Mosaic virus, coding regions were concatenated into frame and then used to generate CAI values.

To calculate normalized CAI, we first used the CAIcal program [31] to obtain a “raw” CAI (rCAI). Next, an “expected neutral CAI” (eCAI) value was calculated by generating 1000 random sequences using similar length, codon composition and GC-content. Normalized CAI values were then compared among different time points and viral lineages using a non-parametric rank test because central tendencies trend varied throughout time more than each time point variance. To obtain our normalized CAI threshold, rCAI/eCAI values were calculated. Values greater than 1 were taken as evidence for codon adaptation to the reference set of codon preferences [31]. Values lower than 1 was taken as evidence that mutational bias are driving codon selection.

### Codon usage tables

The codon usage tables for *Culex pipiens*, *Aedes aegypti*, *Macaca mulata*, *Columba livia* and *Rhipicephalus microplus* were downloaded directly from the publically available Codon Usage Database (www.kazusa.or.jp/codon).

We used 3 in-house codon usage tables that aimed to represent human house-keeping genes, highly expressed human antiviral immune genes, and *Nicotiana tabacum* (tobacco) house-keeping genes. The human codon usage table available on the Kazusa Codon Usage Database was last updated in 2007. Since highly or constitutively expressed genes will manifest greater codon preference biases [1,2,9], we first identified which genes would be potential “targets” for viral codon adaptation. The 3804 identified human house-keeping genes by Eisenberg and Levanon in 2013 [32] were selected for the human house-keeping gene codon usage table. To identify and select highly expressed human antiviral genes, we looked for studies that quantified innate immunity gene expression during infection, specifically differences between mice and human immune responses [33,34], various protein functionalities of the innate immune system during viral infection [35–38], and immune responses during flavivirus infection [39,40]. Ultimately, we selected 25 genes that we believed to represent highly expressed antiviral immune genes. To address the concern that we may be incorrectly comparing a population’s (virus) versus an individual’s genetic components, we crossed-referenced all human coding sequences used with haplotype date from the 1000 Genomes Project to look for single-nucleotide polymorphisms in coding regions of interest [41]. Variants in house-keeping and immune genes did not alter the overall codon frequencies of our generated codon usage tables. All sequences used to generate the human immune table and tobacco house-keeping gene table can be found in S2 Dataset. Comparisons between human tables and between vector tables can be found in S1 Text. Codon usage tables are made available at Github (https://github.com/CaioFreire/CUB).

### WNV phylogenetic inference and Malthusian fitness estimate

First, 790 West Nile Virus complete polyprotein sequences from Genbank were downloaded. Sequences were aligned and curated by coding sequences using Muscle and Geneious respectively. Using FastTree.v2 [42], a maximum-likelihood tree was inferred, and allowed us to partition sequences by previously described lineages [20,43]. Using 61 lineage 2 sequences, the rate of each substitution type under the general reversible model (GTR) substitution model, the proportion of invariant sites (I), and shape parameter of a gamma distribution with four rate categories (Γ_4_) was estimated from the data. Tip times corresponding to the year of virus sampling, a lognormal uncorrelated relaxed clock using continuous quantile parameterization [44], and a GMRF Bayesian Skyride coalescence model [45] was used and run using Beast 1.8.3 [46]. The MCMC analysis was run for 100 million chains to ensure convergence (achieved by a single run, with 10% of the run, discarded as representing “burn in”). A MCC was produced and annotated by the use of TreeAnnotator in the BEAST package. To infer the recent demographic history of WNV in Europe, we employed the Bayesian skyride method [45] and previously described methods to estimate the temporal dynamics of effective population size (Ne.g) of WNV, which approximates the number of infections in time. To reveal the dynamics of viral population size growth, we calculated the Malthusian fitness (*Wm*) for the polyprotein, which was approximated by the ratio of the population size in sequential time points (*Wm* = Ne.gt/Ne.gt−1) [47].

## Results

House-keeping genes are constitutively expressed in all human cells [32,48]; being therefore a likely venue for viral codon adaptation. Moreover, it is fair to assume that human immune genes shape many viral-host interactions and disease severity [33,38]. Usually during a viral acute infection, immune responses are triggered including an increased expression of antiviral genes [35,37,39,49,50]. We did not have any evidence supporting that the host’s tRNA pools alter during viral infection or if the codon preferences of a virus change when cellular immune processes initiate during a recognized viral infection. The immune antiviral genes table was created to provide an alternate human codon table that has different codon usages than that of human housekeeping genes. Between the two human tables, the average difference between the frequencies per thousand bases among triplets is 4.259 (*s*.*d*. = ±4.104) with a max difference of 18.1 (All extended codon table comparisons can be found in Supplementary Data).

**Fig 1:**
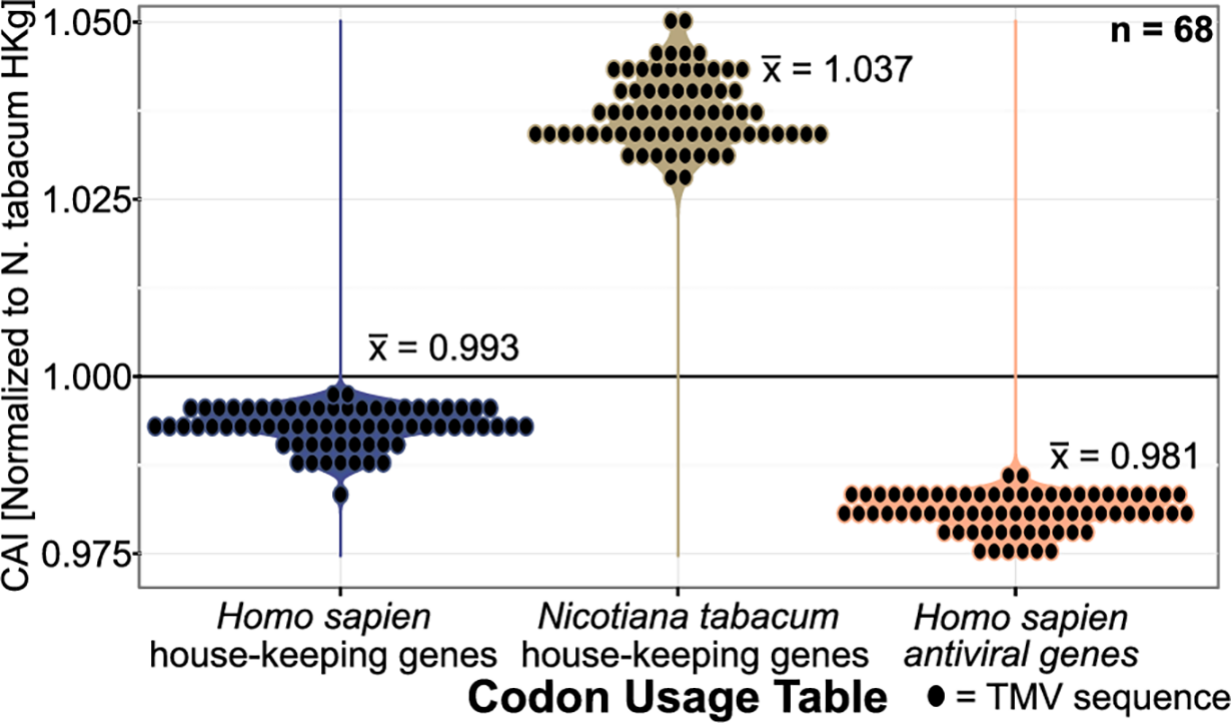
The CAI of Tobacco Mosaic virus to house-keeping genes. A violin dotplot of the CAI of complete Tobacco Mosaic virus (TMV) coding sequences to the codon usages of human house-keeping genes, antiviral/immune genes, and tobacco (*Nicotiana tabacum*) house-keeping genes. Average CAI values for each group are shown.

We first wanted to show that there are measurable and meaningful viral-host adaptations that could be observed using CAI. As a proof of concept for virus-host codon adaptation, we calculated the CAI of Tobacco Mosaic virus (TMV) using complete coding sequences against the codon preferences of tobacco (*Nicotiana tabacum)* housekeeping genes [51,52], TMV’s “natural” host (Fig 1). When TMV sequences were tested against the codon preferences of human genes, there was no evidence for codon adaptation. The average CAI of TMV to tobacco housekeeping genes was 1.037 (s.d. = ±0.005), showing strong evidence for adaptation. On the other hand, TMV’s CAI against human housekeeping and antiviral genes showed no evidence for codon adaptation with an average CAI of 0.993 (s.d. = ±0.003) and 0.981 (s.d. = ±0.003), respectively.

After demonstrating that CAI could be used to measure a virus’ codon adaptation to specific hosts codon choices, we hypothesized that similar relationships could be observed in flaviviruses. They are arthropod-borne viruses that have the ability to infect and replicate in hosts of different phyla. Therefore, versatility in gene expression and protein synthesis is at premium, and certain changes in the viral RNA genome could affect the fitness of the virus in a specific host. Indeed, viral fitness changes relate to dinucleotide frequencies, codon preferences, and codon pair biases [5,14,15,53,54].

**Fig 2:**
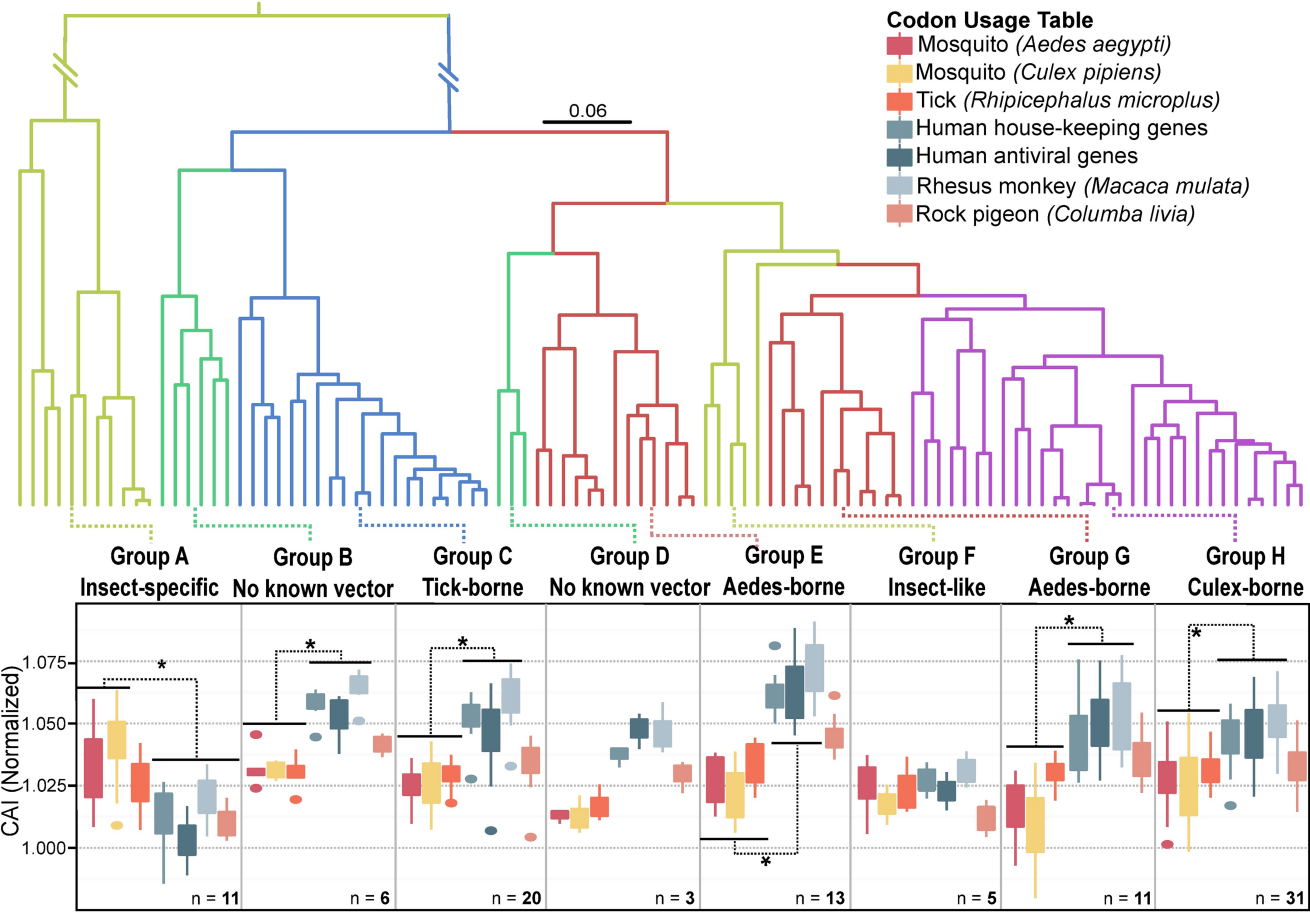
Flavivirus-CAI dynamics to associated species. (A) Bayesian-inferred phylogenetic tree of the complete ORF using nucleotide sequences. All nodes had a posterior probability > 0.9. Taxa were omitted for clarity. Associated viral vectors and vertebrate host groups are colored. The scale bar represents 0.06 mutations per site. (B) Box and whisker plot of CAI for each flavivirus species subgroup. For each group of sequences, the CAI was normalized by length, GC-percentage and amino-acid content. Black lines with asterisks signify CAI values that are significantly different.

Ecology, different virus-host relationships and biogeographical migrations of flavivirus species may explain observed genetic differences among subgroups [13,15,16,55,56]. Phylogenetic studies have acknowledged three groups that reflect the evolutionary and ecological dynamics: tick-borne, mosquito-borne (*Aedes* and *Culex* species), and no known-vector flaviviruses [57–63]. We reconstructed the evolutionary history of the Flavivirus genus based on the complete coding sequences of species used in Moureau *et al*.’s 2015 paper [63], except that we used a nucleotide alignment. As biological and genetic differences are evident between each host/vector subgroup, we calculated the normalized CAI for each sequence in their respective clade against 7 codon usage tables: *i)* human housekeeping, *ii)* highly expressed human antiviral immune genes, *iii) Aedes aegypti*, *iv) Culex pipiens*, *v) Rhipicephalus microplus* (tick), *vi) Macaca mulata* (rhesus monkey) and *vii) Columba livia* (rock dove pigeon) (Fig 2).

Insect-specific groups A includes Cell fusing agent virus (CFAV), Culex flavivirus (CxFV), Aedes flavivirus (AEFV), Kamiti River virus (KMV) and seven others (S1 Fig): all viruses in this group are thought to be vertebrate-incompetent both *in vitro* and *in vivo* [62,64–70]. Both CAI values to human codon and pigeon tables compared to insect vectors were significantly different; with vector CAI values higher (Wilcoxon rank sum test, *p*-values ranged from 1.985x10^-5^ to 0.0457). Although all CAI results for vertebrate CAIs were significantly lower to the *Culex* and *Aedes* tables, we did not find any significant difference between tick and monkey codon adaptation within the group A clade.

The other insect-specific flavivirus group F clade includes 5 species (Chaoyang virus (CHAOV), Donggang virus (DONV), Lammi virus (LAMV), Barkedji virus (BJV) and Ilomantsi virus (ILOV) paralogous to mosquito-borne flaviviruses, supporting a more recent emergence and closer relation to *Aedes* and *Culex*-borne viruses. The general equality across all of the CAIs of group F is different than the other insect-specific group (A), where the CAI of vector species is visibly greater than vertebrate species. The group F species showed no clear relationship between vertebrates and vector species codon usages.

Mosquito-borne and tick-borne groups that have known associations with a vertebrate host revealed a different codon bias. In groups C (tick-borne), E (Yellow fever virus complex), G (Dengue virus complex) and H (West Nile virus complex), viral codon adaptation indexes were significantly higher in mammalian vertebrates than in vector species (Wilcoxon rank sum test, p-values ranged from 5.387x10^-7^ to 0.0192). Additionally, we found that CAI values were on average higher in monkeys than in human genes for vertebrate-associated flavivirus groups, and in some cases differences were significant. A higher CAI average in vertebrates compared to vectors was universal in all vertebrate-associated groups. Interestingly, the group B clade (no known-vector) shared a similar trend to mosquito-borne and tick-borne CAI box plots, where mammalian vertebrate CAIs were significantly higher than vector species (Wilcoxon rank sum test, p-values ranged from 0.002 to 0.008).

The *Culex*-borne flavivirus clade (group H) emerged from *Aedes*-borne flaviviruses thousands of years ago [58,71]. *Culex*-borne species have a life cycle involving primarily avian species and a few species have even been isolated from rodents. For example, the rock dove pigeon (*Columba livia*) is an avian species with prior associations to *Culex*-borne viral infections *i*.*e*. West Nile virus [72]. Like the group G *Aedes*-borne flaviviruses species, CAI values to mammalian vertebrate tables were significantly higher than in mosquito species (Wilcoxon rank sum test, p-values ranged from 5.83x10^-9^ to 1.032x10^-6^). In the case of the rock pigeon, the *Culex*-borne flaviviruses had a lower CAI distribution to pigeons than to mammalian vertebrates (Wilcoxon rank sum test, p-values ranged from 2.201x10^-8^ to 0.000136). Furthermore, *Culex*-borne species had a significantly higher CAI to pigeons than to *Culex pipiens* (Wilcoxon rank sum test, p-value = 0.007) but only averaged higher than *Aedes aegypti* and tick CAI values. Across flavivirus subgroups that are known to harbor viral species with known capability of infecting mammalian vertebrates (B, C, D, E, G, H), the CAI to pigeons rests in the middle of vector and mammalian CAI values.

**Fig 3:**
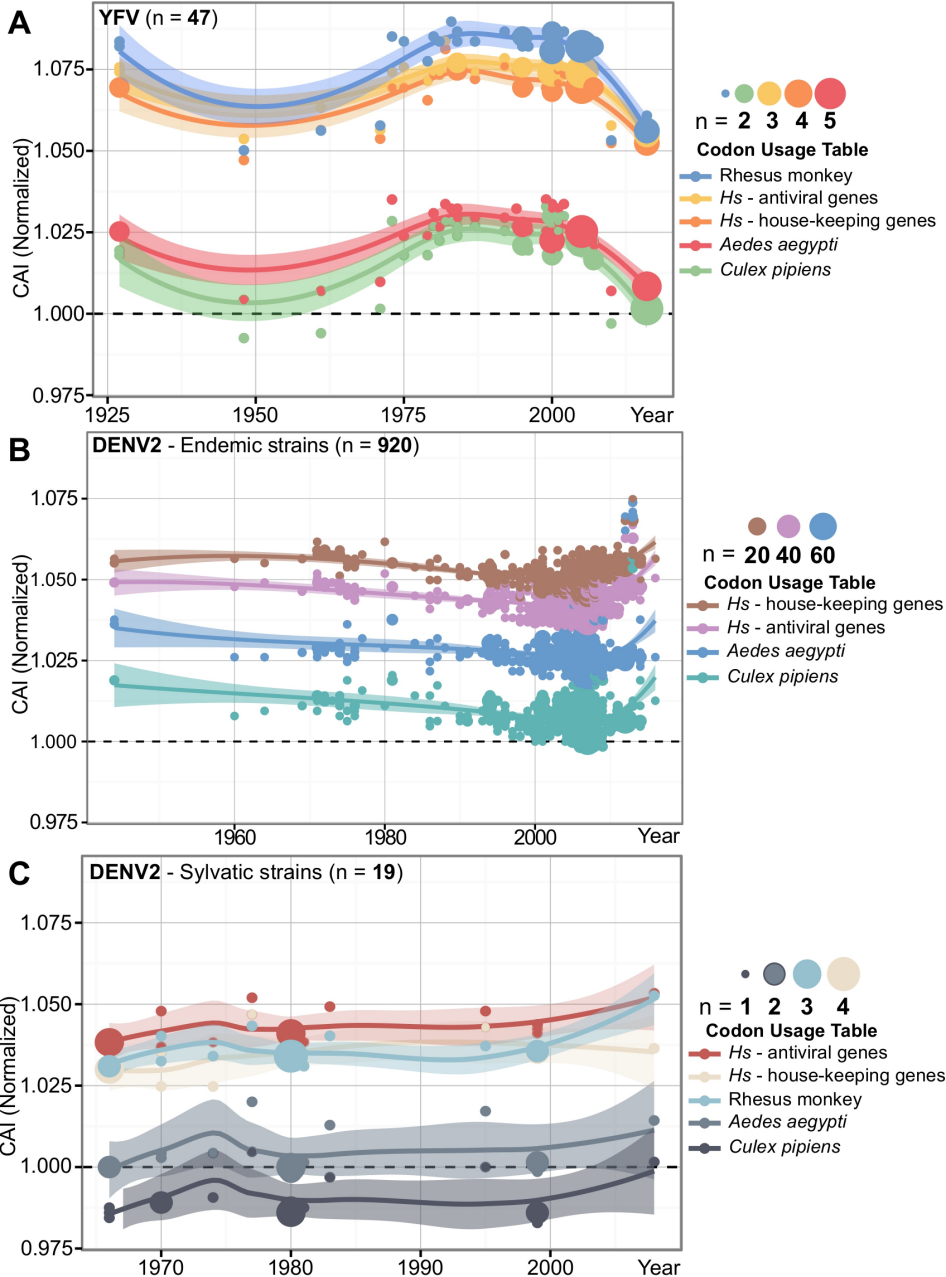
**CAI changes across time for (A) Yellow fever virus, (B) endemic strains of Dengue 2 virus, and (C) sylvatic strains of Dengue 2 virus.** For each codon usage table, the CAI was normalized by length, GC% and amino acid content for each dataset. Area of plot points reflects the density of sequences at a specific coordinate. A trend line was generated using LOESS, a non-parametric regression method, with 0.95 confidence interval shading. For B), CAI data to monkeys was removed for clarity, but was positioned in between the human table trend lines.

After observing CAI differences between the flavivirus genus subgroups, we explored if changes in CAI over time could be seen in specific viral species. Yellow fever virus (YFV, group E) and Dengue 2 virus (DENV-2, group G) are paralogous *Aedes*-borne flaviviruses who boast century-long interactions with human hosts [27,73,74]. Both had large numbers of serially stamped complete ORF sequences available, allowing us to investigate the temporal trends of YFV CAI, and compare differences in the CAI of DENV-2 endemic and sylvatic strains (Fig 3).

To start, we calculated their CAI to human gene codon usage datasets, the Rhesus monkey (*Macaca mulata*) *Culex pipiens* and the *Aedes aegypti* codon usage tables. For the YFV dataset (Fig 3A), the 5 CAI trendlines were highly correlated (Spearman’s rank correlation test, p-value ≤ 2.2x10^-16^, *rho* ranged from 0.977 to 0.999). YFV demonstrated codon adaptation for both vectors and humans across all sampled strains. YFV CAI to Rhesus monkey codon usage was significantly higher than human and vector tables (Wilcoxon rank sum test, p-values ≤ 6.375x10^-7^). We also observed significantly higher CAI for *Aedes aegypti* than in *Culex pipiens* (Wilcoxon rank sum test, p-value = 0.006). Across all three datasets, YFV had a higher CAI to the mammalian codon usage tables, followed by DENV-2 endemic and sylvatic strains respectively.

A higher CAI to its *Aedes*-associated vector species compared to the common *Culex*-borne vector was also true for endemic and sylvatic strains of DENV-2 (Wilcoxon rank sum test, p-values < 1.711x10^-5^) (Fig 3B and 3C). DENV-2 endemic strains, showed highly correlated trend lines across all CAI tests (Spearman’s rank correlation test, p-values < 2.2x10^-16^, *rho* values ranged from 0.875 to 0.996). Since we were more interested in the interplay of DENV-2 endemic strains with humans, particularly when transmission alters between mosquitoes and humans in an urban setting, we removed the CAI data of DENV-2 endemic strains to the Rhesus monkey table. DENV-2 endemic strains showed a significantly higher CAI to housekeeping genes over time (Wilcoxon rank sum test, p-value < 2.2x10^-16^). This is divergent to YFV, where CAI over time was greater to the immune genes codon usage table (Wilcoxon rank sum test, p-value = 5.775x10^-5^).

In DENV-2 sylvatic strains, we saw a different pattern (Fig 3C). DENV-2 sylvatic strains had a higher CAI towards immune genes than they did in house-keeping genes (Wilcoxon rank sum test, p-value = 1.919x10^-5^). Furthermore, sylvatic strains did not show any evidence for codon adaptation towards *Culex pipiens*, as the CAI for all sequences was below 1. Interestingly, codon adaptation to the Rhesus monkey shows an increase in CAI from 1995 to the 2008 sequences, positively correlating with the same increase in CAI that takes place later in DENV-2 endemic strains to human genes from 2003 to 2016 (Spearman’s rank correlation test, p-value ≤ 2.2x10^-16^, *rho* ranged from 0.943 to 0.996). In fact, this same positive correlation was shared in DENV-2 sylvatic strains codon adaptation to *Aedes aegypti*. (Spearman’s rank correlation test, p-value ≤ 2.2x10^-16^, *rho* ranged from 0.946 to 0.995).

After exploring the CAI trends of YFV and DENV-2, both *Aedes*-borne flaviviruses, we then asked if a similar result could be found in a *Culex*-borne flavivirus such as West Nile virus (WNV). WNV is transmitted primarily by the *Culex pipiens* mosquito and can cause severe neurological disease in vertebrates: including humans, horses and birds [75]. Furthermore, the virus has been isolated in every continent except Antarctica [20]. In addition to its vast geographical spread, the WNV species shows high genetic diversity and exists in at least 7 different lineages [17]. WNV lineage 2 (L2) was historically thought as a milder, less pathogenic lineage than its lineage 1 counterpart and was believed to be restricted to sub-Saharan Africa[76,77]. In 2004, the known boundaries of L2 changed when a Hungarian goshawk (*Accipiter gentilis*) had neurological symptoms that were later identified as WNV disease from a L2 strain [78]. L2 was detected in birds, mosquitoes and humans sporadically over the next few years in Eastern European nations [79]. In 2010, a large WNV L2 human outbreak in Greece was reported, with 81 confirmed cases of West Nile neuroinvasive disease [80]. From 2010 to 2014, WNV human outbreaks were reported in Russia, Czech Republic, Italy, Austria, Serbia, Hungary, Belgium and Romania [81].

**Fig 4:**
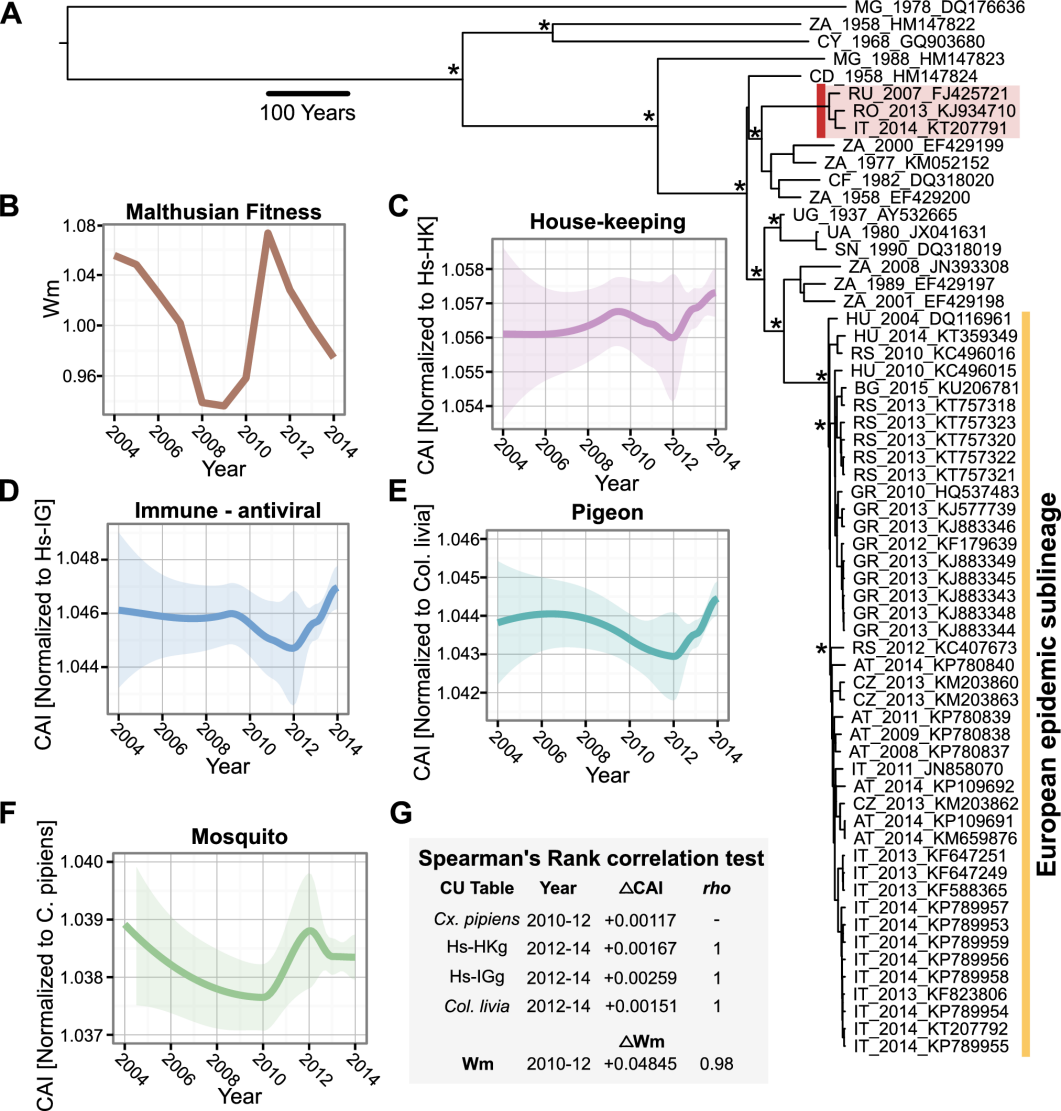
CAI and phylodynamics of West Nile virus lineage 2 sequences. A) Bayesian maximum clade credibility tree representing a time scaled phylogeny of a WNV lineage 2 polyprotein sequences. Bayesian posterior probabilities > 0.9 are marked with an asterisk at major nodes. Averages for the European epidemic lineage (yellow bar) and a 2^nd^ European lineage (highlighted in red) are shown. B) Malthusian fitness (*Wm*) was calculated from 2004–2014, and compared to LOESS trend lines generated from CAI values to the codon usages of C) human house-keeping genes, D) human immune/antiviral genes, E) pigeon (Columba livia) genes, and F) mosquito (*Culex pipiens*) genes. G) The Spearman’s rank correlation test was used to test if there were any correlations between the 2010 to 2012 CAI increase in mosquitoes and the CAI increase in vertebrate species from 2012–2014, as well as Wm. The ΔCAI, Δ*Wm* and *rho* are shown for clarity. # = for all correlations, *p*-values were < 0.05.

With the availability of African and recently sequenced European WNV L2 complete genomes, we investigated the CAI changes of L2 during its recent emergence out of Africa and into Europe. We first show that there were at least two different introductions of L2 into Europe since 2003 (Fig 4A). Interestingly, the epidemic sublineage (n = 43) had a higher CAI than the 3 European sequences outside the clade for vertebrate and mosquito tables (Wilcoxon rank sum test, *p*-values ranged from 0.001 to 0.02). We then calculated the Malthusian fitness (*Wm*) for L2 from 2004 to 2014, a time period that coincided with L2 strains isolated and sequenced in Europe (Fig 4B). *Wm* allows us to observe fitness in a very short period of time, which can show variation in population sizes of a viral species [47].

We then calculated the CAI of L2 sequences against human, mosquito (*Culex pipiens*) and the pigeon (*Columba livia*) codon usage tables. With *Wm*, a measure of relative fitness over time, we were able to compare two independent measurements of viral fitness that span before and during the European WNV L2 epidemic (Fig 4B–4F). We first looked for any trends of *Wm* or CAI from 2004 to 2014 that were similar. We discovered that the trend lines for mosquitoes and *Wm* were correlated (Wilcoxon rank sum test, p = 0.005, *rho* = 0.8). Notably, the joint increase of *Wm* and mosquito CAI during the 2010 to 2012 period was highly correlated (Wilcoxon rank correlation test, p = 0.017, *rho* = 1). Interestingly, an increase of CAI in pigeon and human tables followed in 2012 to 2014 that was highly correlated to the earlier increase in mosquitoes and *Wm* (Spearman’s rank correlation test, p-value ≤ 0.017, *rho* ≥ 0.98). The increase in vector CAI that precedes the increase in human CAI was similar to what we observed in sylvatic DENV-2 CAI to *Aedes aegypti* and rhesus monkeys, followed by a later increase in the CAI of endemic DENV-2 strains in humans.

## Discussion

Observing changes that may be involved in translational efficiency offers a promising avenue for a better comprehension to how some flaviviruses can infect and replicate in human hosts. Codon usage bias studies are inherently common, but they rarely consider the changes in codon adaptation to hosts that occur over time, as we have done here. Although we observed significant differences in the normalized CAI of different viruses, we still lack the knowledge to infer what biological consequences these small changes could cause. Although the critique of sequencing bias, including the scarcity of aged samples, and lack of non-human sequences can limit CAI analyses, we have shown that the results can be informative.

One advantage of calculating the CAI of flaviviruses is the single open-reading frame that codes for around 3400 amino acids. Using such complete polyprotein reading frames, we were able to observe differences in CAI between previously inferred vector and vertebrate phylogenetic groups [5,63,82,83]. In particular, insect-specific groups favored the codon choices of vector species, while mammal-associated flavivirus groups were better adapted to vertebrate codon usage. Insect-specific flaviviruses are thought to sustain their populations in their respective insect vectors in the absence of mammal reservoirs [84], so lower translational efficiency in vertebrates could be expected.

As expected, some of our results could not be connected to some of the known biological aspects of flaviviruses. This could suggest that there may be uncharacterized features of the translational mechanisms at play. For example, the results we obtained using the tick (*Rhipicephalus microplus*) table did not support the biological and epidemiology of tick-borne viruses (Fig 2). Indeed, tick-borne viruses cause dead-end infections in humans [85], so we did not expect CAI to be as high, especially compared to some mosquito-borne viruses that can cause secondary infections in humans [2]. Some tick-borne viruses use rodents as their reservoir hosts, and have associations with other mammalian vertebrates such as dogs, horses, and other large mammals [86], which could have lead to tick-borne viruses having similar codon usages to humans and monkeys. Nevertheless, we did not investigate the CAI of flaviviruses to rodents so far.

The number of insect-specific and arboviral sequences as a whole are rapidly increasing [87,88], which will allow for more in-depth analyses in the future. The capabilities of CAI analysis are limited to the availability of serial-stamped sequencing data, specifically older isolates. Furthermore, the public availability of complete genome sequences has increased as cost for sequencing has decreased and improved over time. Occasionally, we also encountered a limitation as to what inferences we could make on the biology of flaviviruses. For example, in Fig 3C, there is only one complete DENV-2 sylvatic genome isolated in 2008 that suggested a CAI increase in rhesus monkeys and *Aedes aegypti* since the turn of the millennia that was later followed by an increase in human CAI. This was also the case for West Nile virus, where L2 showed an increase in Malthusian fitness and *Culex pipiens* prior to an increase in human CAI (Fig 4B–4G). Although there was not enough DENV-2 sylvatic sequences to calculate Malthusian fitness, comparing Malthusian fitness and CAI could help elucidate evolutionary dynamics of an emerging virus [89].

Crucially, just one sequence could be enough to make statistical inferences regarding changes in codon adaptation to a specific host. This is due to the fact that every one of the 3400 codons in the genome is counted, and tested against 500 random sequences with the same length and amino acid content to determine if codon changes are adaptive or random. Therefore, arguably every flavivirus sequence included in our analysis provides substantial amount of data.

During an ongoing epidemic where sufficient sampling and sequencing have been generated, we could use these sequences to observe changes in fitness of a virus that could coincide with fluctuations of CAI to different hosts. This was the case for WNV L2, for which we had access to 46 complete polyprotein sequences sampled in Europe since 2008. Our phylogenetic inference supported results from previous works that suggest multiple entries of WNV into Europe [22,90–92]. Interestingly, we found that a small group of European sequences (highlighted in red in Fig 4) had significantly lower CAI values in mammals than the epidemic sublineage composed of the rest of the European L2 sequences. This could be explained by sampling bias or possibly that these strains lacked the translational efficiency to spread with the same force as the other European strains (Fig 4A). We lack *in vivo* experimental data, but we can hypothesize how our results can shed light on the complexity of viral translational adaptation over time in different vectors and hosts.

Coupling CAI analysis with *in vivo* and *in vitro* viral titer quantification, and functional genetics could improve the understanding of the phenotypic impact of synonymous changes, such as in the cases of the comparison between the African and Brazilian strains of the Zika virus [10]. In a similar manner, a possible follow-up study would be to test *in vivo/in vitro* kinetics (see [93] for an example) for endemic and sylvatic strains of DENV2 from the same sampling location. There are many gaps in our understanding of how synonymous and non-synonymous changes lead to functional changes in viral replication, virulence and host adaptation [94]. Nevertheless, temporal analyses of CAI can be informative in better identifying time-stamped sequences with non-synonymous changes that could provide an advantage in different hosts. In sum, we hope more studies will identify housekeeping genes for other peridomestic species capable of transmitting zoonotic viruses, such as flavivirus vectors and *Culex*-associated migratory birds.

## Supporting information

S1 Dataset Data used to generate figures. The dataset is divided by the data used for each figure. In each tab, accession numbers and normalized CAI values are included.
(XLSX)

S2 Dataset Codon usage table genes. Genes used in the creating of in-house codon usage tables, including tobacco house-keeping genes and human immune/antiviral genes.
(XLSX)

S1 Fig Bayesian-inferred phylogenetic tree of the complete ORF using nucleotide sequences. Taxon are included and all nodes had a posterior probability > 0.9. Associated viral vectors and vertebrate host groups are colored. The scale bar represents 0.06 mutations per site.
(EPS)

S1 Text Codon usage table comparisons using *codcmp*. Using the *codcmp* program from the EMBOSS package, raw outputs from relevant comparisons between human, tobacco, and vector species codon usage tables are shown.
(TXT)
